# Imaging the Kidney with an Unconventional Scanning Electron Microscopy Technique: Analysis of the Subpodocyte Space in Diabetic Mice

**DOI:** 10.3390/ijms23031699

**Published:** 2022-02-01

**Authors:** Sara Conti, Giuseppe Remuzzi, Ariela Benigni, Susanna Tomasoni

**Affiliations:** Istituto di Ricerche Farmacologiche Mario Negri IRCCS, 24126 Bergamo, Italy; giuseppe.remuzzi@marionegri.it (G.R.); ariela.benigni@marionegri.it (A.B.); susanna.tomasoni@marionegri.it (S.T.)

**Keywords:** scanning electron microscopy, subpodocyte space, diabetic nephropathy

## Abstract

Transmission electron microscopy (TEM) remains the gold standard for renal histopathological diagnoses, given its higher resolving power, compared with light microscopy. However, it imposes several limitations on pathologists, including longer sample preparation time and a small observation area. To overcome these, we introduced a scanning electron microscopy (SEM) technique for imaging resin-embedded semi-thin sections of renal tissue. We developed a rapid tissue preparation protocol for experimental models and human biopsies which, alongside SEM digital imaging acquisition of secondary electrons (SE–SEM), enables fast electron microscopy examination, with a resolution similar to that achieved by TEM. We used this unconventional SEM imaging approach to investigate the subpodocyte space (SPS) in BTBR *ob*/*ob* mice with type 2 diabetes. Analysis of semi-thin sections with secondary electrons revealed that the SPS had expanded in volume and covered large areas of the glomerular basement membrane, forming wide spaces between the podocyte body and the underlying filtering membrane. Our results show that SE–SEM is a valuable tool for imaging the kidney at the ultrastructural level, filling the magnification gap between light microscopy and TEM, and reveal that in diabetic mice, the SPS is larger than in normal controls, which is associated with podocyte damage and impaired kidney function.

## 1. Introduction

Since the first transmission electron microscope (TEM) was developed by Ernst Ruska in 1931 [1,2], it has been considered an essential tool for the ultrastructural analysis of healthy, diseased, and experimental tissue in renal histopathology, and has contributed significantly to our understanding of renal diseases [3,4,5]. Compared with light microscopy, TEM achieves higher resolving power due to the shorter wavelength of the electron beam it uses [6]. However, although the crucial role that TEM plays in diagnostic renal pathology is undisputed, its use is not without challenges and imposes a number of limitations on pathologists that must be considered. Low penetration of the electron beam limits samples to a thickness of about 100 nm, making sample preparation for TEM a complex and very time-consuming process [7]. TEM examination, in addition to being cumbersome, can also be frustrating because pathologists can examine only a small fraction of the tissue, due to the visual obstructions caused by TEM grids, at the expense of collecting information on the structural organisation of the whole sample. Therefore, a system that can reduce sample preparation time and allow imaging at a high resolution for a large volume of samples is needed.

Scanning electron microscopy (SEM) is usually used to obtain 3D imaging of the surface of the sample [8,9], in contrast with TEM, which offers information on the inner structure of the sample. Nevertheless, emerging evidence points to the usefulness of backscattered electron scanning microscopy (BSE–SEM) imaging in ultrastructural analyses of large sample fields. In that regard, several protocols for processing tissues for BSE–SEM are described [10,11,12]. These protocols are essentially created ad hoc to obtain better results with backscattered electrons (BSEs) and often recommend the en bloc staining of renal tissue prior to it being embedded in resin, to improve contrast and the signal-to-noise ratio, meaning that some of these protocols cannot be applied to retrospective studies or experimental or human biopsies that are already embedded in resin. 

Therefore, in this study, we modified existing BSE–SEM methods to develop a simple, fast and versatile method, the so-called SE–SEM, which can be applied to all tissues fixed for conventional TEM and make it possible to obtain TEM-like images from resin-embedded semi-thin sections using secondary electrons (SEs). To confirm the validity and potential of our method in ultrastructural tissue analysis, we applied this technique to the examination of podocyte alterations, focusing particularly on subpodocyte space (SPS) changes in the BTBR *ob*/*ob* mice, a well-established model of diabetic nephropathy in type 2 diabetes [13]. Our group has already shown, using TEM, that there is an increase in SPS in an experimental model of progressive glomerular disease [14]. To quantify SPS extension in our earlier study, we had to acquire different sections for each glomerulus by TEM and then mount all images to create a unique high-resolution image of the entire glomerulus, onto which we superimposed a digital grid for morphometric analysis [14], making SPS analysis a complex and very time-consuming process. 

In this study, we applied our SE–SEM method to validate its great potential and usefulness in ultrastructural tissue analysis.

## 2. Results

### 2.1. TEM-like Images from Resin-Embedded Semi-Thin Sections Using SE–SEM

**Figure 1:**
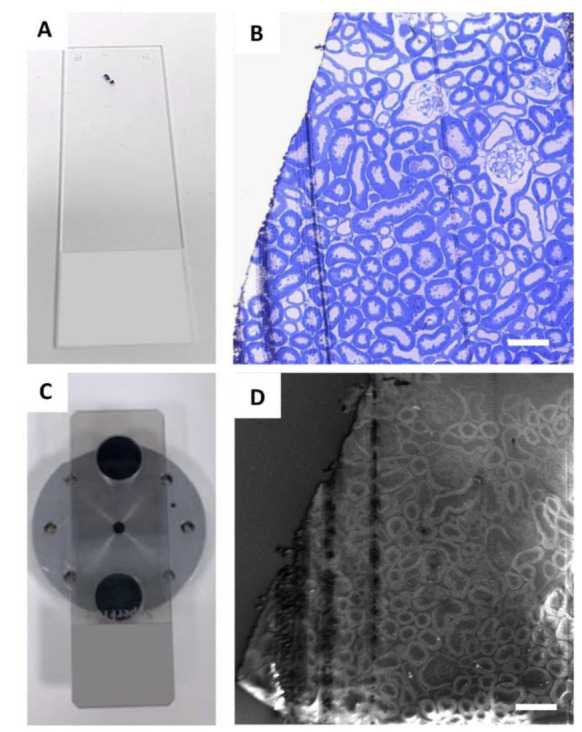
Identification of glomeruli for SE–SEM analysis: (**A**) regions of interest were identified on semi-thin sections, collected on a standard glass slide, and stained with toluidine blue (**B**) and observed with light microscopy; (**C**) for SE–SEM analysis, sections were collected on carbon-coated slides, mounted on an SEM specimen holder and (**D**) observed with SEM, with secondary electrons. Scale bars: 100 µm.

Regions of interest were identified on toluidine blue semi-thin renal sections (Figure 1A,B) and then collected on a carbon-coated slice (Figure 1C) for observation with an ultra-high-resolution field-emission SEM with a Gemini column (Figure 1D).

**Figure 2:**
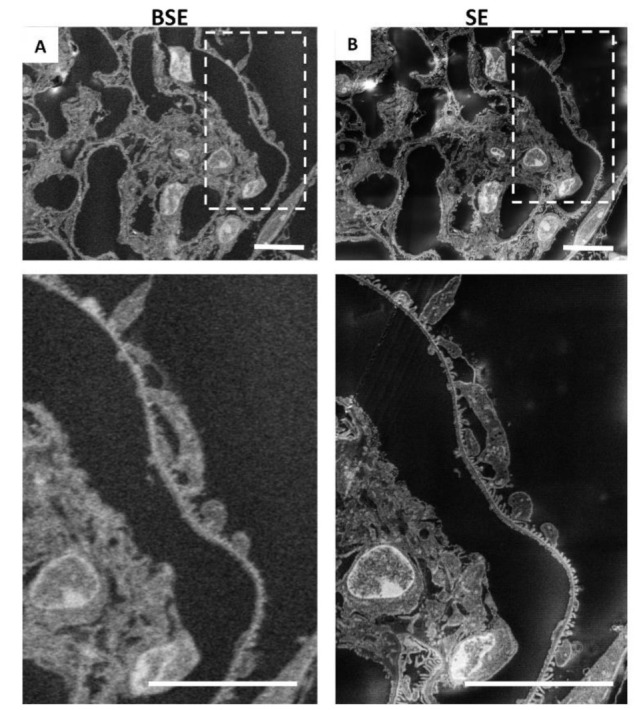
Resolution obtained by secondary electrons is higher than that achieved by backscattered electrons: (**A**) representative image of renal ultrastructure taken with SEM with backscattered electrons or (**B**) with secondary electrons. Insets (lower panels) show a high-power view of the same cells. Scale bars: 10 µm.

SEM scans a focused and high-energy beam of electrons over the surface of a sample to create an image. As the electrons interact with the sample, they produce secondary electrons (SEs) and backscattered electrons (BSEs), and here, we analysed the sample with both types of electrons, to find the acquisition ideal condition (Figure 2A,B). Secondary electrons, the most widely used signal, have lower energy than the backscattered electrons (<50 eV) [15]. Consequently, SEs can only escape from the top few nanometres of the surface of a sample. The signal from secondary electrons tends to be highly localised at the point of impact of the primary electron beam, making it possible to collect images of the sample surface with a resolution of below 1 nm [16]. In contrast, BSEs have much higher energy than SEs, and they emerge from deeper locations within the specimen [15]. Consequently, the resolution of BSE images is lower than that of SE images [17,18]. As shown in Figure 2, we confirmed that the best results, in terms of resolution, acquisition time, and signal-to-noise ratio, were obtained with SEs (Figure 2B), compared with BSEs (Figure 2A). 

To obtain the TEM-like images of the renal tissue, we used an acceleration voltage of 2 kV, with a 120 µm aperture, in “high current” mode, which increases the active probe current. The working distance was set up at 8 mm. Glass slides were sputter coated with a different layer of carbon (Supplemental Figure S1A, see Supplementary Materials), and the best results in terms of resolution and signal-to-noise ratio were obtained with a coating of 60 nm of carbon (Supplemental Figure S1B), compared with 150 nm (Supplemental Figure S1C). During the initial observation steps, to significantly reduce the charging effects, the glomeruli were identified in the section with a low scan speed of 1 and at a low resolution (1024 × 768) (Figure 1D). Later, to obtain the final high-resolution image, each glomerulus was analysed with a scan speed of 4–5 and with a line averaging noise reduction of about 1 min. The pixel dimensions for a recorded image were 3072 × 2304 pixels. The contrast and brightness of the images were adjusted directly at the SEM during the acquisition. To perform the morphometric quantifications, we preferred to use the original SE–SEM images (Supplemental Figure S2A), although, depending on the pathologist’s preferences, it is also possible to reverse the grayscale and obtain TEM-like image colours (Supplemental Figure S2B). 

**Figure 3:**
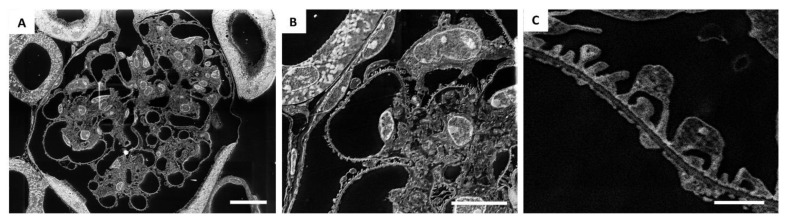
Application of SE–SEM method to image the kidney from the structural to the ultrastructural level: (**A**) representative SE–SEM images of the entire glomerulus at a very high resolution; (**B**,**C**) SE–SEM imaging provides detailed images of the renal ultrastructure at different magnifications. Scale bar represents 10 µm for (**A**,**B**), and 1 µm for (**C**). Panel (**B**) is an enlargement of panel (**A**).

Notably, as shown in Figure 3, with the SE–SEM technique, it was possible to obtain images of the entire glomerulus at a very high resolution (Figure 3A), not comparable to those obtained using light microscopy. In addition to the overview of the entire glomerular tuft, SE–SEM imaging provided detailed images of the renal ultrastructure at different magnifications (Figure 3B,C) very quickly and easily. 

**Figure 4:**
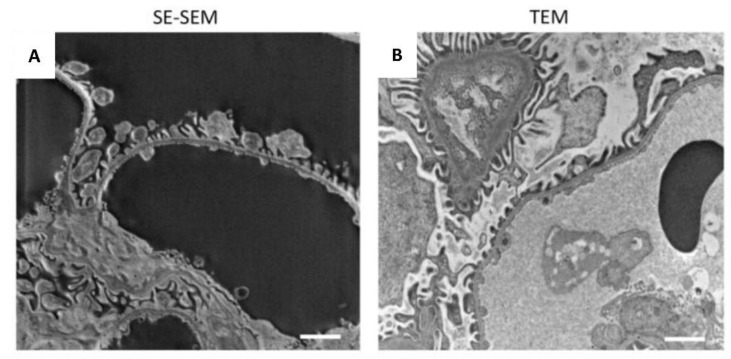
Comparison of the SE–SEM image with TEM image: (**A**) representative image of renal ultrastructure taken with SE–SEM or (**B**) with TEM. Scale bars: 1 µm.

We were then extremely impressed by the realisation that the glomerular architecture images taken at higher magnification provided a resolution of details similar to those in TEM images (Figure 3C and Figure 4). SE–SEM images of the glomerular filtration barrier were impressively similar to TEM images taken at the same magnification (Figure 4A,B). As shown in Figure 4A, the three major components of the filtration barrier—the fenestrated endothelial cell, the glomerular basement membrane, and the podocyte with their foot processes—were clearly visible in the SE–SEM photomicrograph and, notably, even the fine structure of glomerular basement membrane with its lamina densa and lamina rara.

**Figure 5:**
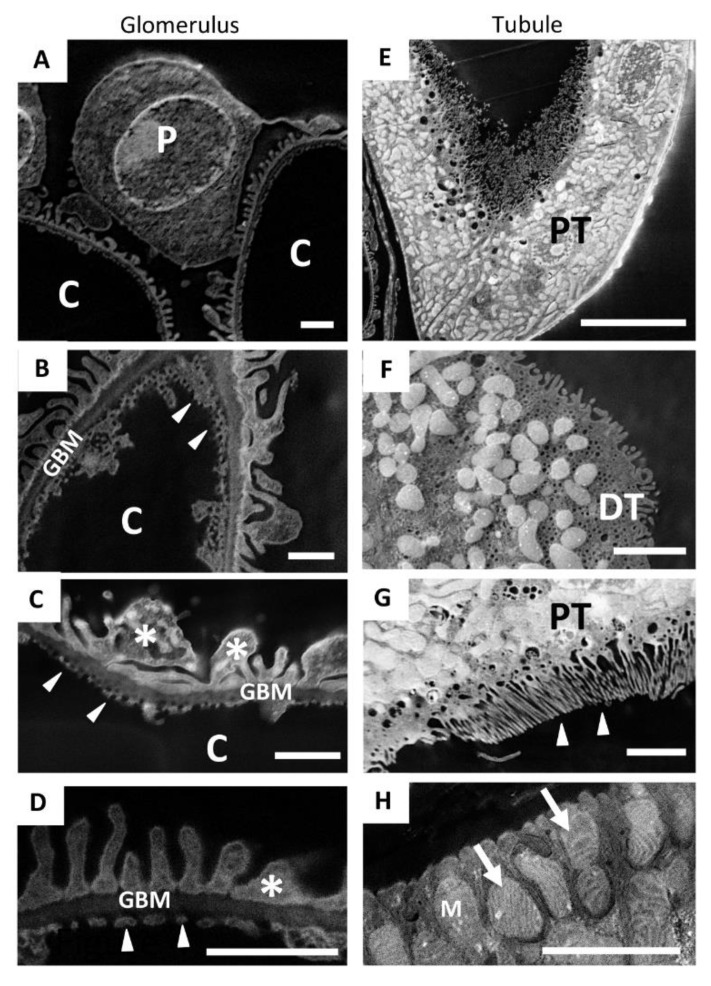
SE–SEM method makes it possible to recognise the fine ultrastructure of the different renal compartments: (**A**–**D**) representative SE–SEM images of the components of the glomerular filtration barrier, such as (**B**–**D**) fenestrated endothelium (arrowheads) and (**D**) podocyte foot processes (asterisks); (**E**,**H**) SE–SEM images of the tubular compartment; (**F**) a distal tubule and (**E**) a proximal tubule, with (**G**) its luminal brush border (arrowheads); (**H**) the complex organisation of lamellar cristae (arrows) within the mitochondria of tubular cells observed with SE–SEM. Scale bars: 1 µm for (**A**–**D**); 10 µm for (**E**) and 2 µm for (**F**–**H**). Abbreviations: P, podocyte; C, capillary lumen; GBM, glomerular basement membrane; PT, proximal tubule; DT, distal tubule; M, mitochondria.

Even the smallest structures of the kidney tissue were clearly observable in detail when observed using SE–SEM, with a resolution similar to TEM. As shown in Figure 5, our SEM method provided two-dimensional, high-resolution images of the components of the glomerular filtration barrier (Figure 5A), such as fenestrated endothelium (Figure 5B–D) and podocyte foot processes (Figure 5C,D), which were extremely impressive. In the tubular compartment (Figure 5E–H) the SE–SEM imaging approach showed the fine structure of the distal (Figure 5F) and proximal tubules (Figure 5E), with its luminal brush border (Figure 5G) and, much to our surprise, even the complex organisation of lamellar cristae within mitochondria of tubular cells (Figure 5H). 

### 2.2. Characterisation of Kidney Functional Parameters of BTBR ob/ob Mice 

Systemic and laboratory parameters measured in BTBR *ob*/*ob* diabetic and BTBR wild-type (WT) control mice are shown in Table 1. Consistent with previous data [19,20,21], BTBR *ob*/*ob* mice exhibited severe hyperglycaemia and increases in diuresis, compared with WT mice. Urinary albumin excretion and urine creatinine levels were enhanced in diabetic mice, compared with BTBR WT mice [22], while levels of systolic blood pressure were comparable to those of control mice, as previously reported [23].

### 2.3. SE–SEM Ultrastructural Analysis in BTBR ob/ob Mice

**Figure 6:**
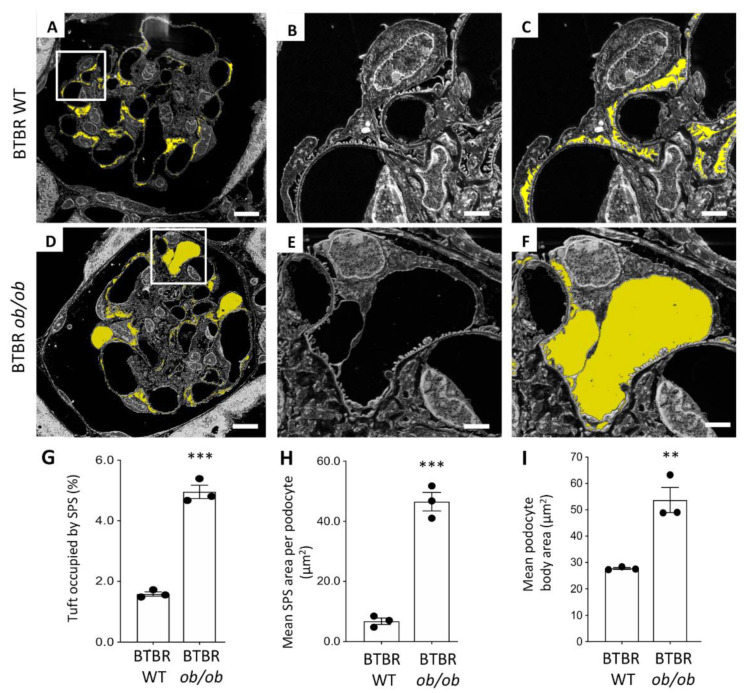
Morphological changes in the subpodocyte space and in podocytes documented using the SE–SEM method on BTBR *ob*/*ob* mice: (**A**–**C**) segmentation of the subpodocyte space (SPS) area on images acquired from BTBR WT and (**D**–**F**) BTBR *ob*/*ob* mice; (**G**) quantification of SPS area expressed as percentage, (**H**) mean SPS area per podocyte, and (**I**) quantification of the podocyte body area, in BTBR WT and BTBR *ob*/*ob* mice at 19 weeks of age. The SPS is highlighted in yellow. (n = 3 animals for each group, 5 glomeruli for each animal). *** *p* < 0.0001, ** *p* < 0.01. Scale bars: 10 µm, (**A**,**D**); 2 µm, (**B**–**F**). Data are expressed as mean ± SEM.

The high resolving power achieved by the SE–SEM approach prompted us to investigate ultrastructural changes in podocytes and SPS in BTBR *ob*/*ob* mice. We analysed the ultrastructure of the SPS through the segmentation of the SPS area on images acquired from resin-embedded semi-thin sections (Figure 6A–F). In diabetic mice, SPS covered large areas of the glomerular basement membrane, forming wider spaces between the podocyte body and the underlying filtering membrane (Figure 6D–F), compared with what was observed in WT mice (Figure 6A–C). The morphometric analysis performed on SE–SEM photomicrographs revealed that there was a significant increase in the SPS, which had expanded in volume and occupied a larger percentage of the glomerular tuft in compared to what is observed in healthy animals (Figure 6G). 

In diabetic mice, the mean SPS area per podocyte was significantly higher than in BTBR WT mice (Figure 6H) and was almost seven times higher than in the control WT group.

Since it is known that the enlargement of SPS increases the mechanical forces exerted on podocytes by water filtration and induces podocyte detachment from the glomerular basement membrane [14] and that BTBR *ob*/*ob* mice were characterised by a significant loss of podocytes [19,20,24,25], we then identified and analysed the ultrastructure of these cells in BTBR *ob*/*ob* mice. Consistent with previous data [26], diabetic BTBR *ob*/*ob* mice were characterised by hypertrophic changes in podocytes. As shown in Figure 6I, the mean area occupied by the podocyte body was significantly higher in BTBR *ob*/*ob* than in WT mice (averaging 27.72 µm^2^ and 53.72 µm^2^, respectively, in BTBR WT and BTBR *ob*/*ob* mice, *p* < 0.01), indicating the hypertrophy of the few remaining podocytes in diabetic mice. 

**Figure 7:**
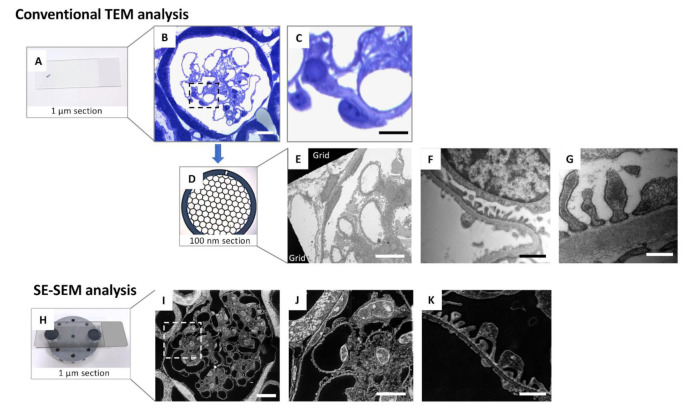
A simplified comparison between conventional TEM analysis and the SE–SEM method in renal ultrastructural examination: (**A**–**G**) conventional TEM tissue preparation protocols, including (**A**–**C**) the identification of the glomerulus using light microscopy on toluidine blue stained 1 µm sections and then (**D**) the collection of 100 nm sections on copper TEM grids for (**E**–**G**) observation with TEM; (**H**–**K**) SE–SEM method protocol, including (**H**) collection of 1 µm sections on carbon-coated slice and (**I**–**K)** observation by an ultra-high-resolution field-emission SEM of the semi-thin (1 µm) section. The SE–SEM method allows the observation of the sample, from a structural to ultrastructural level, in the same section. Scale bars: 10 µm for (**B**,**C**,**E**,**I**,**J**), and 1 µm for (**F**,**K**), 500 nm for (**G**). Panel (**C**,**J**) were enlargements of panel (**B**,**I**), respectively.

Most importantly, compared with a previous evaluation of SPS using TEM in our earlier study [14], the application of SE–SEM dramatically reduced the duration of the entire process by almost 70%, making ultrastructural analysis of the SPS a faster and easier process (Figure 7).

## 3. Discussion

Here, we develop an unconventional scanning electron microscopy approach that enables kidney imaging at the ultrastructural level, providing a high level of precision and accuracy while saving time and resources. Our SE–SEM method is a fast and valuable tool that fills the magnification gap between light and transmission electron microscopy (Figure 7). 

Previous studies have focused on the use of backscattered electrons or ad hoc tissue processing protocols, limiting the application of these methods. To the best of our knowledge, this is the first report to combine conventional TEM tissue preparation protocols with SEM digital imaging acquisition of secondary electrons of semi-thin sections to investigate the glomerular ultrastructural alterations induced by diabetic nephropathy. SE–SEM speeds electron microscopy examination up considerably, with a far higher resolution than light microscopy and similar to that achieved by TEM (Figure 7), and can also be applied to retrospective studies or biopsies that are already embedded in resin. 

Although our findings support the claim that SE–SEM evaluation is a powerful tool for analysing renal biopsies, the crucial role that TEM plays in diagnostic renal pathology is undisputed [27,28,29], and there is no doubt that SE–SEM does not achieve the resolution power of TEM. However, SE–SEM is a valuable alternative to TEM when high-resolution imaging of a large volume of the sample is needed or when the pathologist does not need to reach maximum magnifications for the purposes of the study. TEM and SE–SEM should be viewed not as competitive techniques or alternatives to each other but rather as complementary methods for achieving the same goal.

One of the great advantages of our system is the notable reduction in time, for both sample preparation and analysis. Compared with conventional TEM analysis, the entire sample preparation time for SE–SEM is reduced to the time necessary to collect the semi-thin sections on a carbon-coated glass slice (Figure 7). Moreover, the use of the same section for all magnification levels avoids the risk of missing an interesting field observed in the toluidine blue section during the further sample cut necessary to obtain the ultra-thin sections for TEM analysis. Furthermore, the SE detector is very fast and easy to use. Given that we have obtained ultrastructural information from 1 µm sections, our SE–SEM method also opens up new perspectives regarding the possibility of performing a three-dimensional reconstruction of the entire sample, with a TEM-like resolution, through the very straightforward collection of 1 µm serial sections on carbon-coated slices and the speedy acquisition of serial photomicrographs through SEM secondary electrons. 

In this report, we applied our method to the study of SPS in an experimental model of diabetic nephropathy, but this is a good example of the potential this approach has. The high resolving power achieved by SE–SEM was perfect for visualising and segmenting the SPS with high accuracy and precision, as well as clearly identifying glomerular cells and the podocytes, based on their morphological features. This suggests that our SEM imaging approach could also be used in all studies that explore the relationship between the size and numbers of glomerular resident cells and renal diseases [30,31,32]. 

High glucose has been reported to induce cellular hypertrophy in podocytes [33,34], and it is known that podocyte hypertrophy precedes apoptosis under experimental diabetic conditions [26]. The hypertrophic changes in podocytes documented using the SE–SEM method in diabetic mice are consistent with the data in the literature obtained with other methods, confirming the reliability of our processing and imaging protocol and making us confident about our SE–SEM observations. In addition to the hypertrophic changes in podocytes, our morphometric data document the SPS rearrangement in diabetic BTBR *ob*/*ob* mice. Given the complexity of the very heterogeneous 3D spatial organisation of the SPS, further studies are needed to assess the exact mechanism underlying SPS changes in experimental diabetic nephropathy.

However, the most important observation we make is based on the fact that the SE–SEM method has allowed us to observe a phenomenon such as the increase in SPS with extreme ease and rapidity, paving the way for future studies.

It is our hope that our results will encourage pathologists to consider scanning electron microscopy, and in particular, the SE–SEM method, as a valuable tool that can provide insights that complement the diagnostic approach provided by TEM and that can be used in routine experimental and clinical pathology.

## 4. Materials and Methods

### 4.1. Experimental Design

This study was performed on kidney samples from male BTBR *ob*/*ob* and BTBR WT mice obtained from Jackson Laboratories (Bar Harbour, ME, USA). BTBR *ob*/*ob* mice (n = 3 mice/group) developed albuminuria at 10 weeks of age. BTBR WT mice served as controls (n = 3). Animals were maintained in a temperature-controlled room regulated with a 12 h light–dark cycle and had free access to water and food. At sacrifice at 19 weeks of age, kidneys were obtained and processed for morphological evaluation through perfusion fixation, as previously described [14,35].

### 4.2. Biochemical Parameters

Blood glucose levels were assessed with a reflectance meter (OneTouch UltraEasy, LifeScan, Milan, Italy). Urinary albumin excretion was measured with the ELISA test using the Bethyl test kit (catalogues E101, A90-134A, and A90-134P, Bethyl Laboratories Inc., Montgomery, TX, USA). Urinary creatinine concentration was measured using an enzymatic method with Miura one auto-analyser (I.S.E. S.r.l. Rome, Italy). Systolic blood pressure was measured with a computerised tail-cuff system in conscious mice (BP-2000 Blood Pressure Analysis System, Visitech System; Apex, White Oak, NC, USA).

### 4.3. Tissue Fixation and Processing for SE–SEM Ultrastructural Analysis 

We modified the BSE–SEM method, described by Reichelt et al. [10], to obtain high-resolution images from semi-thin sections of kidney tissue processed for conventional TEM. The kidney fragments were placed in a glass vial for overnight fixation in freshly prepared 2.5% glutaraldehyde in 0.1 M cacodylate buffer pH 7.4. The tissue was washed 3 × 10 min with 0.1 M cacodylate buffer, and then, the buffer solution was replaced with 1% osmium tetroxide in cacodylate buffer for 1 h of postfixation. After postfixation, specimens were dehydrated through a series of ascending grades of ethanol concentrations (10% ETOH × 5 min, 50% × 10 min, 70% × 10 min, 90% × 15 min, and finally, 100% × 15 min) and were rinsed with propylene oxide for 15 min. The propylene oxide was replaced with 50% epoxy resin (Agar Scientific Ltd, Stansted, UK.) and 50% propylene oxide mixture (*v*/*v*, 1 mL per sample), and the tissue was immersed for 4 h. This mixture was freshly prepared in advance, at room temperature. The intermediate solution was replaced with epoxy resin for 12 h (overnight) for tissue infiltration, and each fragment was gently transferred to the tip of the embedding mould. Moulds were placed in the oven at 60°C for 72 h to obtain polymerised blocks. This critical step required careful control of working temperature (58–60 °C).

### 4.4. Sections Preparation for SE–SEM Ultrastructural Analysis 

In the ultramicrotome chamber, the excess resin was removed from the face of the block using a single-edge carbon steel razor blade to obtain a truncated pyramid with a trapezoidal base. Semi-thin sections (1 µm) were cut using a diamond knife (Diatome histology 8 mm) with UMC ultramicrotome (Leica Biosystem; Buffalo Grove, IL, USA). Sections were collected on a glass loop and transferred to a drop of water on a standard glass slide. The slide was heated on a heating plate, and a drop of 1% toluidine blue was added (in 5% borax, filtered, Sigma-Aldrich, St. Louis, MO, USA) to stain the plate for 10 s. Sections were gently washed with distilled water (dH_2_O) and were examined under a light microscope to identify areas of interest and confirm the presence of glomeruli (Figure 1A,B). The next section was collected on carbon-coated glass slides. Glass slides were previously sputter coated (EMS150R ES PLUS, Leica Biosystem; Buffalo Grove, IL, USA) with a different layer of carbon, to evaluate the ideal layer thickness (60 and 150 nm) (Supplemental Figure S1A). Semi-thin sections (1 µm) were cut with UMC ultramicrotome and collected on carbon-coated glass slides and dried on a heat plate at about 50° for 5 min. Finally, sections were stained with UAR (diluted 4x in dH_2_O), an alternative to uranyl acetate stains—which are environmentally problematic due to their radioactivity [36]—for 1 min and lead citrate for 1 min each, to improve the contrast in the SE–SEM image and reduce charging effects during SEM analysis. Sections were then gently washed with distilled water (dH_2_O) and dried on a heating plate at about 50°.

### 4.5. SE–SEM Imaging Acquisition 

Images were acquired using an ultra-high-resolution field-emission SEM with a Gemini column (SEM 1540XB CrossBeam, Carl Zeiss; Oberkochen, Germany). The carbon-coated slide with sections was mounted on an SEM specimen holder using two aluminium pin stubs (12.5 mm diameter), positioned at the ends of the slide using carbon conductive mounting tabs, i.e., double-sided adhesive discs, which maintain the sample’s mechanical stability (Figure 1C). The sections were analysed using SEM (Figure 1D), with different detectors (BSE, In Lens, and SE) and with different acceleration voltage settings and aperture and working distance, to identify the ideal parameters.

### 4.6. Ultrastructure Morphometrical Analysis on SE–SEM Images

Five glomeruli per animal were analysed. Images of the glomerular capillary tuft were taken randomly using SEM. The contrast and brightness of the images were adjusted using ImageJ software (version 1.51, National Institutes of Health, Bethesda, MD, USA) and GIMP (GNU Image Manipulation Program, Gimp 2.8, The GIMP Development Team) to reverse the grayscale and obtain TEM-like image colours. All ultrastructure morphometrical analyses were performed in 5 glomeruli for each animal, on digitised images using the Morpholio Trace software (version 2.0, Apple, Cupertino, CA, USA). Briefly, the fraction of the tuft area occupied by SPS was manually segmented for each glomerular section image (Supplemental Figure S3). This procedure was made faster and easier by using an iPad Pro 12.9-inch (4th generation, Apple, Cupertino, CA, USA) as an interactive display tablet, with a digital pen (Apple pencil second generation, Apple, Cupertino, CA, USA), which made it possible to segment the area of interest with high accuracy and precision. The glomerular tuft occupied by SPS was expressed as the percentage ratio of the SPS surface area over the total area of the capillary tuft [37]. 

### 4.7. Statistics

Results are mean ± SEM or SD. Data analysis was performed using Prism Software (GraphPad Software, Inc., La Jolla, CA). All data were analysed using the Student’s *t* test for unpaired data. Statistical significance was defined as a *p* value less than 0.05.

## Figures and Tables

**Table 1:** Systemic parameters measured in BTBR WT and BTBR *ob*/*ob* mice.

Groups	Blood Glucose (mg/dL)	Diuresis (mL/24 h)	SBP (mmHg)	UAE (µg/24 h)	U Creat (mg/dL)
BTBR WT	104.67 ± 12.99	1.10 ± 0.25	102.67 ± 6.17	15.86 ± 2.01	1.31 ± 0.39
BTBR *ob*/*ob* + vehicle	531.33 ± 35.18 **	21.33 ± 9.22	91.00 ± 5.51	2396 ± 1548	41.71 ± 3.45 **

Values are expressed as mean ± SD. ** *p* < 0.01 vs. BTBR WT; n = 3 animals for each group. Abbreviations: SBP, systolic blood pressure; UAE, urinary albumin excretion; U Creat, urinary creatinine.

## Data Availability

Not applicable.

## Supplementary Materials

The following are available online at https://www.mdpi.com/article/10.3390/ijms23031699/s1.
