# Supplementary material for: Imaging the Kidney with an Unconventional Scanning Electron Microscopy Technique: Analysis of the Subpodocyte Space in Diabetic Mice
Source: Int J Mol Sci. 2022 Feb 1;23(3):1699. doi: 10.3390/ijms23031699 (PMC8836024; doi:10.3390/ijms23031699)
Supplement: Supplementary file 1 [file ijms-23-01699-s001.zip › ijms-1551017-supplementary.pdf]

International Journal of
*Molecular Sciences*

MDPI

*Supplementary Materials*

*Article*

# Imaging the Kidney with an Unconventional Scanning Electron Microscopy Technique: Analysis of the Subpodocyte Space in Diabetic Mice

**Sara Conti *, Giuseppe Remuzzi, Ariela Benigni, and Susanna Tomasoni**

Istituto di Ricerche Farmacologiche Mario Negri IRCCS, 24126 Bergamo, Italy;
giuseppe.remuzzi@marionegri.it (G.R.); ariela.benigni@marionegri.it (A.B.);
susanna.tomasoni@marionegri.it (S.T.)
* Correspondence: sara.conti@marionegri.it

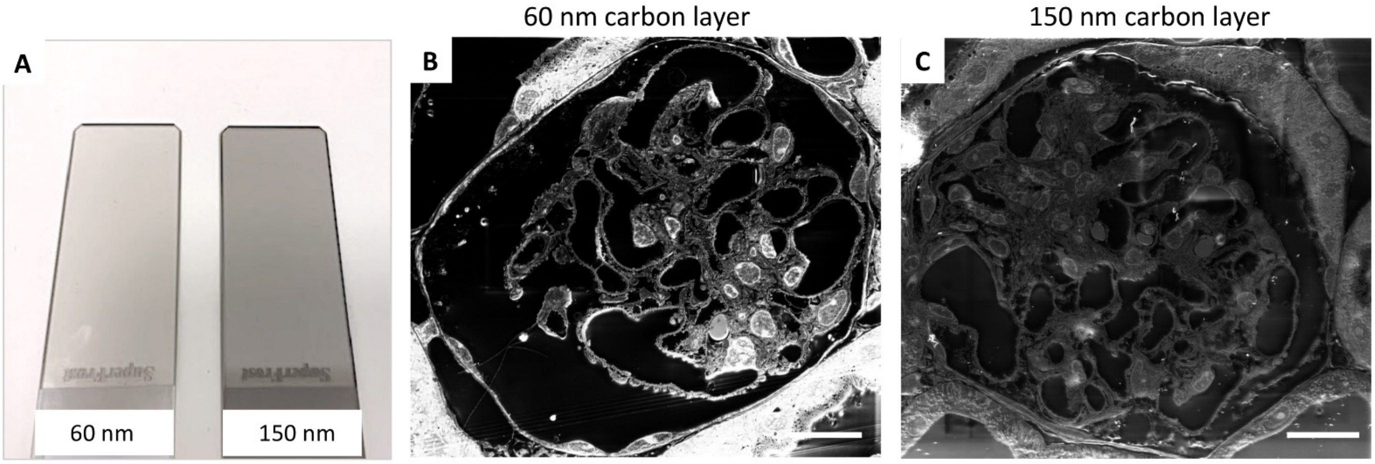


**Figure S1.** Comparison of different thicknesses of carbon coating. (**A**) Glass slides were sputter-coated with a different layer of carbon. (**B**) Representative SE-SEM micrographs acquired from glass slides coated with 60 nm and (**C**) with 150 nm of carbon. Scale bars: 100 µm.

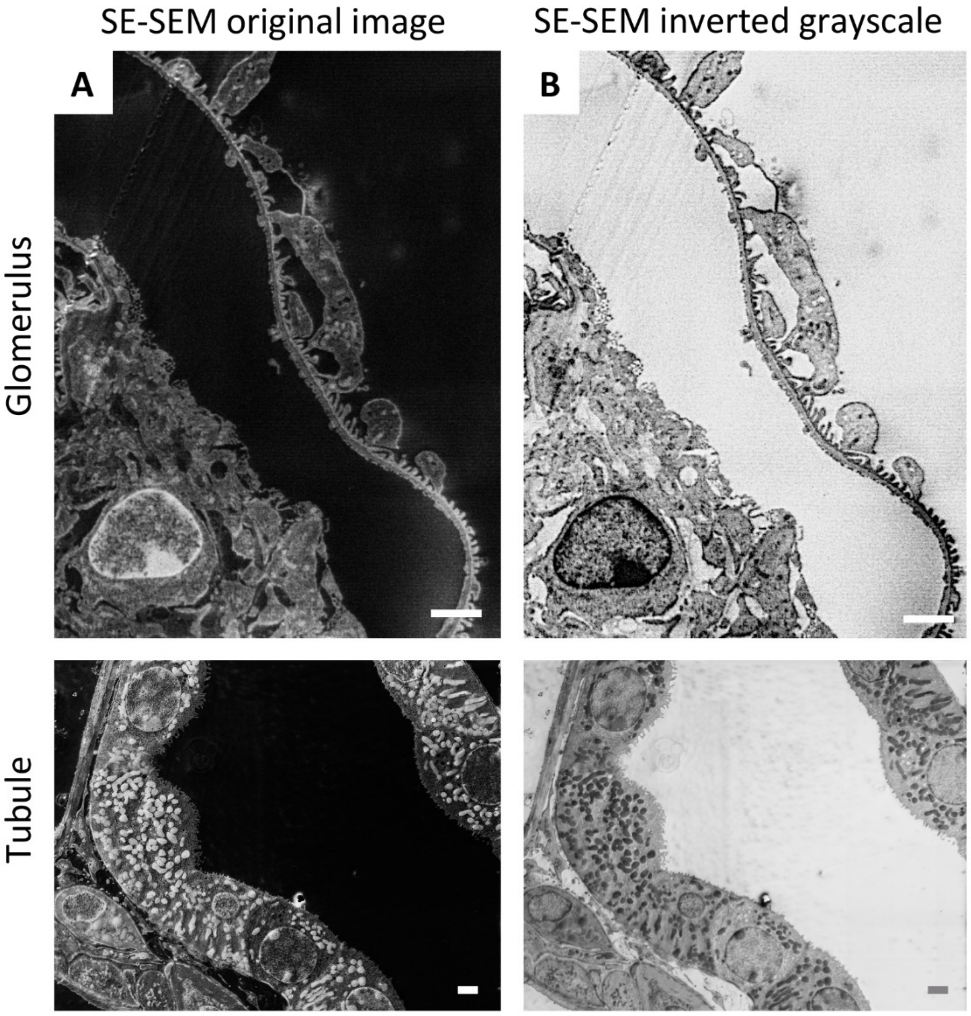


**Figure S2.** Comparison of the original SE-SEM images with TEM-like SE-SEM image colours. (**A**) Original SE-SEM micrographs of the glomerulus (upper panel) and the tubule (lower panel). (**B**) TEM-like images colour of the glomerulus (upper panel) and the tubule (lower panel), obtained by reversing the SE-SEM images grayscale. Scale bars: 2 µm.

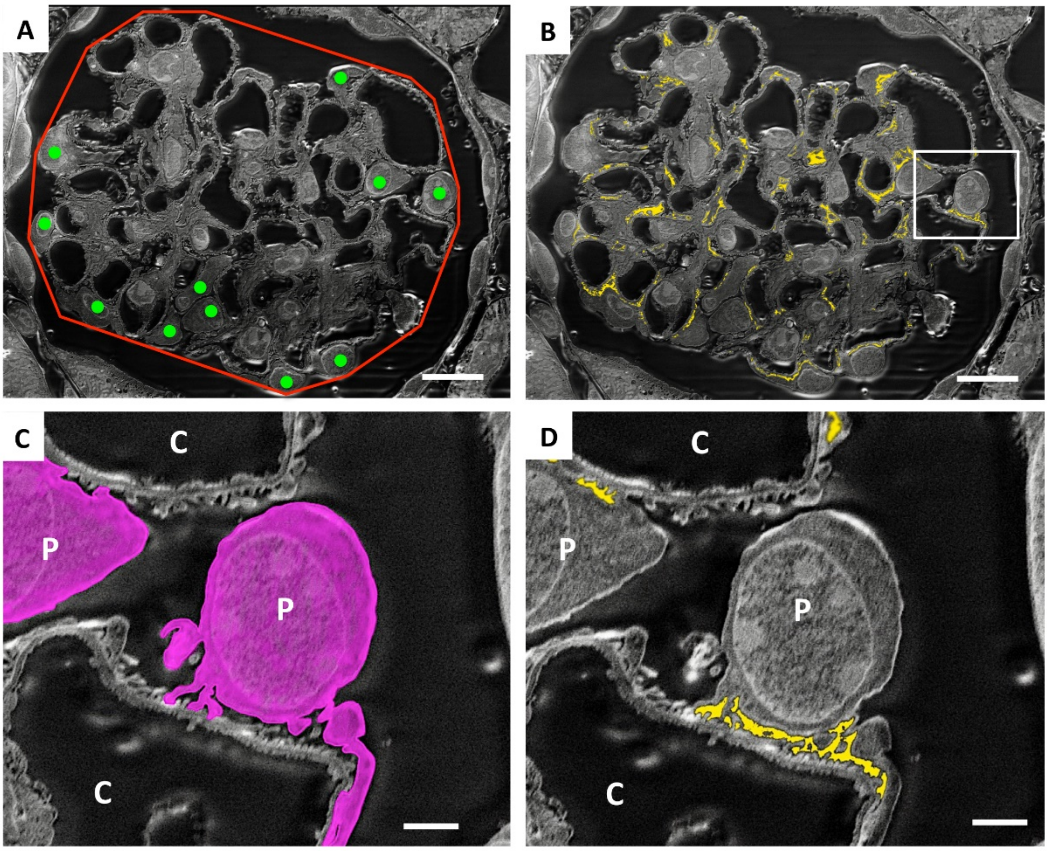


**Figure S3.** The SE-SEM image segmentation method. (**A**) Glomerular area was estimated by outlining the outer polygon of the glomerular capillary tuft and podocytes were clearly identified based on their morphological features (green dots). (**B**) The high resolving power achieved by SE-SEM was perfect for visualising and segmenting the SPS with high accuracy and precision. (**C**) Segmentation of the podocyte body (highlighted in pink) and (**D**) of the SPS (highlighted in yellow), on images acquired from BTBR WT mice. Scale bars: 10 μm for A and B; 2 μm for C and D. Abbreviations: P, podocyte; C, capillary lumen.
